# Detection of EGFR mutations in plasma and biopsies from non-small cell lung cancer patients by allele-specific PCR assays

**DOI:** 10.1186/1471-2407-14-294

**Published:** 2014-04-28

**Authors:** Britta Weber, Peter Meldgaard, Henrik Hager, Lin Wu, Wen Wei, Julie Tsai, Azza Khalil, Ebba Nexo, Boe S Sorensen

**Affiliations:** 1 Department of Clinical Biochemistry, Aarhus University Hospital, Norrebrogade 44, Aarhus 8000, Denmark; 2 Department of Oncology, Aarhus University Hospital, Aarhus, Denmark; 3 Department of Pathology, Aarhus University Hospital, Aarhus, Denmark; 4 Department of Genomics and Oncology, Roche Molecular Systems, Inc, Pleasanton, CA, USA

**Keywords:** EGFR (Epidermal growth factor receptor), Plasma DNA, Erlotinib, Lung cancer

## Abstract

**Background:**

Lung cancer patients with mutations in the epidermal growth factor receptor (EGFR) are primary candidates for EGFR-targeted therapy. Reliable analyses of such mutations have previously been possible only in tumour tissue. Here, we demonstrate that mutations can be detected in plasma samples with allele-specific PCR assays.

**Methods:**

Pairs of the diagnostic biopsy and plasma obtained just prior to start of erlotinib treatment were collected from 199 patients with adenocarcinoma of non-small-cell lung cancer. DNA from both sample types was isolated and examined for the presence of mutations in exons 18–21 of the EGFR gene, employing the cobas® EGFR Tissue Test and cobas® EGFR Blood Test (in development, Roche Molecular Systems, Inc., CA, USA).

**Results:**

Test results were obtained in all 199 (100%) plasma samples and 196/199 (98%) of the biopsies. EGFR-activating mutations were identified in 24/199 (12%) plasma samples and 28/196 (14%) biopsy samples, and 17/196 (9%) matched pairs contained the same mutation. Six EGFR mutations were present only in plasma samples but not in the biopsy samples. The overall concordance of the EGFR gene mutations detected in plasma and biopsy tissue was 179/196 (91%) (kappa value: 0.621).

**Conclusion:**

Mutational analysis of the EGFR gene in plasma samples is feasible with allele-specific PCR assays and represents a non-invasive supplement to biopsy analysis.

**Trial registration:**

M-20080012 from March 10, 2008 and reported to ClinicalTrials.gov: NCT00815971.

## Background

Activating mutations in the tyrosine kinase domain of the EGFR gene in tumour tissue predict clinical response of advanced non-small-cell lung cancer (NSCLC) to tyrosine kinase inhibitors (TKI’s) such as gefitinib and erlotinib [1-4]. However, obtaining adequate tumour tissue from patients with NSCLC for molecular analysis can be challenging. First, the biopsies are often small, and the number of tumour cells retrieved may be too low to allow such analysis. Second, the biopsy may not be representative of the total burden of mutated cells, especially in patients with metastases. A third problem is that genetic changes may take place during the interval between removal of the biopsy and start of TKI treatment, especially in patients receiving chemotherapy or radiotherapy [5]. Previous reports showed that EGFR mutations can be detected in patient’s serum or plasma [6-11], but the success rates for detecting EGFR in blood appear to vary according to the technology used [7-12].

The cobas® EGFR Tissue and Blood tests are allele-specific PCR assays designed to detect EGFR gene mutations in exons 18–21. Here, we evaluated these allele-specific PCR assays’ ability to detect EGFR mutations in plasma samples removed just prior to start of treatment with erlotinib, as compared to the results obtained for the diagnostic biopsy.

## Methods

### Patients

A cohort of 199 patients with advanced adenocarcinoma (or mixed tumours with an adenocarcinoma component) treated at the Department of Oncology, University Hospital of Aarhus, from October 2008 to October 2011, were used for the study. During 2008–2011, the standard treatment for all patients with adenocarcinoma of the lung was carboplatine (AUC 5) i.v. and oral vinorelbine 60–80 mg/kg as first line and erlotinib as second line (Table 1). Archived plasma samples from blood taken prior to erlotinib treatment (within 2 days) were used for EGFR testing, and the diagnostic biopsies were retrieved for tissue testing. Only 196 diagnostic biopsy samples were tested because no tumour cells could be identified in two biopsy samples, and one biopsy did not contain a sufficient quality of DNA for the mutation analysis.

**Table 1:** Patients and tumour characteristics for 199 lung cancer patients scheduled for treatment with erlotinib

	**Number**	**Percent**
**Gender**		
Male	101	51
Female	98	49
**Ethnicity**		
Asian	1	<1
Caucasian	198	>99
**Smoking history**		
Current smoker	64	32
Former smoker	118	59
Never smoker	17	9
ECOG performance status*		
0	26	13
1	99	49
2	63	32
3	11	6
**Tumour type**		
Adenocarcinoma	190	95
Adenosquamous carcinoma	9	5
**Tumour stage**		
IIA + IIB	2	1
IIIA + IIIB	29	15
IV	168	84
**Erlotinib therapy**		
First-line	22	11
Second-line	156	78
Third-line	16	8
Fourth-line	5	3
**Age** (years, mean (range))	64 (33–87)	

*Eastern Cooperative Oncology Group scale to assess how the disease affects the daily living abilities of the patient.

Patient characteristics were obtained for all 199 patients (Table 1).

### Trial registration

The project was approved by the Central Denmark Region Committees on Biomedical Research Ethics (M-20080012) and reported to ClinicalTrials.gov (NCT00815971). Written informed consent for participation in the study was obtained from all participants.

### Tumour and plasma samples

In all, 197 of 199 patients had tumour cells in the diagnostic biopsy (99%). In 92 of 197 patients (47%), the diagnostic tissue was from a fine-needle aspiration biopsy (cytology), whereas in the other 105 patients (53%), the diagnostic tissue consisted of formalin-fixed paraffin-embedded tissue from a gross-needle biopsy or from a surgical specimen. All samples were re-evaluated by the same pathologist to confirm the original diagnosis and classified using the 2004 World Health Organisation (WHO) classification. If more than one block or several smears were available from the same biopsy, the one that contained the highest number of tumour cells was used for testing. The pathologist evaluated the percentage of tumour cells. The tumour cell content was below 30% in most samples (64.5%) and below 10% in 12.7% of the samples. Most biopsies (102) were from the primary tumour, but for 22 and 75 patients, mutation detection was performed on biopsies from lymph node metastasis or distant metastasis, respectively. Blood samples were collected in collection tubes (©Terumo, Europe NV) containing EDTA prior to the erlotinib treatment (within 2 days). Centrifugation was performed at 1000 rpm for 15 min, and plasma was removed and stored at -80°C.

The plasma samples were collected at a median of 10.5 months after the diagnostic biopsy had been taken, and our results are therefore not a direct comparison of the presence of mutated EGFR in biopsies and plasma collected at the same time.

### DNA extraction from paraffin-embedded tissue sections

Five slices were cut from the paraffin-embedded blocks. The two outermost sections were stained with haematoxylin/eosin. If tumour cells could be identified in both sections, the three middle slices were used for DNA extraction. Sections were deparaffinised by submersion in xylene and rehydrated with ethanol. DNA was extracted using the QIAamp DNA FFPE Tissue Kit (Qiagen, Germany) according to the manufacturer’s protocol.

### DNA extraction from archived cytological slides

Manual macro-dissection was performed in 4% of the samples when the tumour cells were clustered in small areas. The selected cells were carefully removed from the slide surface. In the remaining 96% of the samples, all of the cells were taken. The slides were rinsed in 96% ethanol. PBS (phosphate buffered saline) was added on the smear surface before the cells were gently scraped from the slide. DNA was extracted using the QIAamp DNA Mini Kit (Qiagen, Germany) according to the manufacturer’s protocol. The resulting DNA was measured with a Nanodrop UV–vis Spectrophotometer and then diluted with DNA SD (sample diluent) to 2 ng/μL concentration.

### DNA extraction from plasma

For each testing 2 mL of the plasma was used. In the cobas® DNA Sample Preparation kit, Proteinase K, WBI (wash buffer I) and WBI (wash buffer II) were prepared according to the manufacturer’s instructions. The plasma was mixed with 250 μL Proteinase K and 2 mL DNA PBB (binding buffer) and incubated at room temperature for 30 minutes. Then 500 μL isopropanol was mixed with the lysate and transferred into the High Pure Extender Assembly. The High Pure Extender Assemblies were centrifuged at 4000 × g for 1 min. The extenders were removed from the filters; the filters were placed in new collection tubes and washed with WBI and WBII according the manufacturer’s instructions. The DNA was eluted in 100 μL DNA EB (elution buffer).

### Mutation analysis

The cobas® EGFR Tissue Test was used for mutation detection. The test is designed to detect G719A/C/S in exon 18; 29 deletions in exon 19; S768I, T790M and 5 insertions in exon 20; and L858R in exon 21. In addition to mutations detected in the tissue test, the cobas EGFR Blood Test also detects L861Q in exon 21. For the FFPET samples, 50 ng genomic DNA was used for each PCR reaction, and for the plasma samples, 25 μL of the DNA eluate was used for each PCR reaction. The cobas® 4800 SR2 System Software v2.0 and EGFR Analysis Package Software v1.0 were used for tissue analysis. For the blood testing, the EGFR Blood Analysis Package Software (in development) was used. The cobas® EGFR tissue and blood tests were provided by Roche Molecular Systems, Inc. free of charge as the result of research collaboration.

### Statistical analysis

False-positive and false-negative rates could not be determined, as no reference or gold standard has been defined for EGFR mutation analysis. To test the difference between paired samples (biopsy and plasma), McNemar’s test was used. The difference was considered statistically significant when p < 0.05. The statistical software SPSS (18) for Windows; SPSS Inc., Chicago, IL, USA) was used for the calculations.

## Results

The EGFR mutation status was determined in all plasma samples (199/199), giving a success rate of 100%. In the 199 biopsy samples from the same patients, the success rate was 98% (196/199). One tissue sample did not contain sufficient DNA, and other two tissue samples did not contain any tumour cells. Interestingly, in one of these three patients, an L858R mutation was found in the plasma DNA. Among 199 advanced adenocarcinoma patients, 24/199 (12%) were EGFR-mutation positive in plasma and 28/196 (14%) were EGFR-mutation positive in tumour DNA.

A comparison of EGFR mutations in plasma and tumour DNA is shown in Table 2. The overall concordance of EGFR mutation status in plasma and tumour biopsy was 91% (179/196). In all, 17/196 (9%) patients had the same EGFR mutations in plasma as in their original diagnostic biopsy, and 162/196 (82%) patients remained mutation negative. There was no statistically significant difference between the frequency of samples with mutations detected in plasma and biopsy DNA (p = 0.332) as tested by McNemar’s test. In this study, a difference in EGFR mutation status in plasma and original biopsy was observed in 17 of 196 (9%) patients. Of the 23 patients that were EGFR-mutation positive in plasma DNA, 6 were positive in the plasma only, and of the 28 patients that were EGFR mutation positive in tumour DNA, 11 were mutation positive in the tumour only. These differences could reflect the limitation of assay technology with circulating cell-free DNA in the plasma, tumour heterogeneity, and/or the effect of chemotherapy.

**Table 2:** EGFR mutations in paired samples of plasma DNA and biopsy samples (n = 199)

	**Plasma DNA**		
**Biopsy DNA**	**Mutated**	**Wild-type**	**Total**
Mutated	17	11	28
Wild-type	6	162	168
Not classified	1	2	3
Total	24	175	199

Deletions in exon 19 were the most common mutation and were found in 75% and 82% of all mutations in the biopsy and plasma samples, respectively. Point mutations in exon 21 (L858R) represented 21% and 18% of the mutations found in tumour tissue and plasma samples, respectively. A single exon 20 insertion was found in a biopsy sample, but none were observed in plasma DNA.

We found the same mutation rate in cytology and histology specimens (15% vs. 14%), supporting the notion that both specimens are suitable for DNA extraction and EGFR mutation analysis. For the patients with mutations in the blood but not in the biopsy, the tumour cell count in the biopsy was similar to that in the rest of the tumour samples in the study (between 10% and 50%). The fraction of cytology samples was 50% (three cytology and three histology samples) and resembled the rest of the tumour samples in the study.

**Figure 1:**
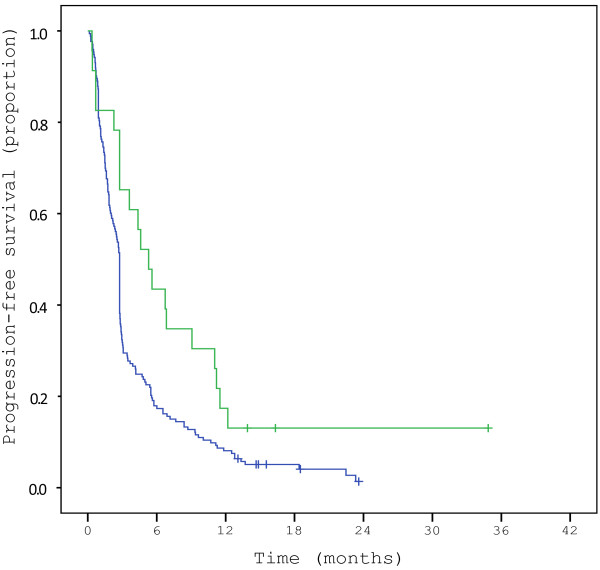
**Kaplan-Meyer curve showing progression-free survival (PFS) of patients with (green line) or without (blue line) activating EGFR mutations identified in the plasma DNA.** The group with activating EGFR mutations has a significantly longer PFS than the group without these mutations, with a p-value of 0.01.

Patients with activating EGFR mutations in plasma DNA had a longer progression-free survival than patients without these mutations (p = 0.01) as illustrated in Figure 1. Median progression-free survival was 5.7 months in the patients with EGFR mutations versus 2.8 months in the patients without these mutations.

## Discussion

In this study, we show that it is feasible to detect EGFR mutations in plasma from patients with advanced lung cancer. Among 199 patients, there were 24 that carried EGFR mutations that could be identified in plasma samples taken just prior to start of treatment with the EGFR-directed drug erlotinib.

The primary objective of this study was to explore the use of a newly developed method for identification of EGFR mutations in plasma. In addition, we compared the mutation status in plasma to the mutation status in the original diagnosis biopsy from the same patients. Plasma samples were collected at a median of 10.5 months after the biopsy was obtained. Thus our results do not represent a direct comparison of the presence of mutated EGFR in biopsies and plasma collected at the same time. Despite this, several interesting findings were observed. First, the allele-specific PCR assay is robust and produced test results for 100% of the plasma samples. Second, for 17 patients whose EGFR mutations were detected in both the diagnostic biopsy and the plasma sample identical mutations were found in both the diagnostic biopsy and plasma sample collected prior to their 2^nd^ line treatment.

Interestingly, EGFR mutations were detected only in the biopsy in 11 patients or only in the plasma in 6 patients. The results could reflect the sensitivity limitation of allele-specific PCR or biological differences in blood when patients progressed from first-line chemotherapy. A mutation found only in the diagnostic biopsy but not in plasma removed several months later may reflect the disappearance of the mutated cells, and an investigation of biopsies and plasma samples taken at the same time could elucidate this. Most of our patients (78%) were primarily treated with conventional chemotherapy and radiotherapy until progression and then erlotinib treatment was started. Currently, it is unknown whether biomarker status changes after exposure to chemotherapy or on progression, but a recent study by Bai et al. demonstrates that chemotherapy might reduce EGFR mutation frequency in both plasma and biopsy samples [5]. That insufficient mutated DNA is released to the plasma to allow detection is another possibility in patients with mutations in biopsies but not in plasma. Also, the occurrence of mutated DNA in plasma despite a wild-type result in the diagnostic biopsy is of interest. This may reflect the appearance of mutated cells between the taking of the diagnostic biopsy and the plasma sample but may also reflect the presence of mutated metastases not reflected in the diagnostic biopsy. Another explanation could be that some tumours contain several clones of which only some contain mutations in the EGFR gene and that the biopsy was taken in a clone without EGFR mutation. So far the therapeutic consequences of mutations present only in the plasma remain unknown, and the seven patients identified in this study are too few to allow us to analyse this question.

In our study, we demonstrated that patients with activating EGFR mutations in plasma DNA have a significantly longer progression-free survival than observed in the group without these mutations, with a median progression-free survival of 5.7 vs. 2.8 months, respectively. This suggests that mutation detection in plasma DNA has clinical utility, but larger studies are required to clarify this.

Previous studies have compared the presence of mutated EGFR in plasma and in biopsies. These studies are all relatively small, and comparisons are hampered by variation in design, use of either plasma or serum, and in differences in methods used for the detection of EGFR mutations [5-9,11,12]. The concordance between EGFR status in tumour and plasma/serum samples varies from 58% to 93% in these studies. This variance might be explained by different sensitivities of the methods used. Most of these studies were retrospective and based on only a few selected patients. In the IPASS study, serum and tissue samples were collected before any therapy, but the concordance was only 66% [8]. A recent study found a concordance rate between tumour and serum DNA of 92%, but this study was based on only 22 patients [12]. Thus, the concordance of 91% in our large cohort of unselected patients is high compared to other studies.

A discordance of EGFR mutations in primary tumours and corresponding metastases has been reported [13] as well as intratumoural heterogeneity. Only a few studies have investigated the influence of this heterogeneity on treatment response, but a recent study suggests that the mutation status in the metastasis has a high impact on treatment outcome [13]. Even though this heterogeneity is known in many cancers and especially in NSCLC, a clinical decision is often based on only one biopsy. As the blood mirrors the entire tumour burden, the use of plasma DNA for EGFR mutation might be more precise and informative. It is well known that mutations causing TKI resistance that cannot be detected in the pre-treatment specimen in patients responding to TKIs may be detected later when the patients relapse [10]. Tumour genotyping by using plasma DNA isolated immediately before and during treatment may therefore be more informative than tumour genotyping of the diagnostic biopsy and may prove useful to monitor therapy response and/or disease progression.

## Conclusion

We demonstrated the feasibility of measuring EGFR mutations in plasma. Our results warrant further studies in order to clarify whether plasma can replace biopsies for the detection of EGFR mutations and to what extent serial measurements of mutations in plasma may help the clinician in choosing the optimal therapy for the patient.

## Competing interests

The cobas® EGFR tissue and blood tests were provided by Roche Molecular Systems, Inc. free of charge as the result of research collaboration.

## Authors’ contributions

BW analysed tumour DNA, participated in the design of the study, and was involved with the statistics and drafting of the manuscript. PM was involved with the design and coordination of the study and helped to draft the manuscript. HH did the pathological examinations of the tumour samples. LW participated in the design of the study and evaluated the mutation results. WW participated in the design of the study and analysed the mutation data. JT participated in designing the study and in analysing the data. AK was involved in the design of the study and in performing the statistical analyses. EN was involved in the design of the study and helped to draft the manuscript. BSS participated in the design and coordination of the study and helped to draft the manuscript. All authors read and approved the final manuscript.

## Pre-publication history

The pre-publication history for this paper can be accessed here:

http://www.biomedcentral.com/1471-2407/14/294/prepub
